# The Relationship Between Teachers' Classroom English Proficiency and Their Teaching Self-Efficacy in an English Medium Instruction Context

**DOI:** 10.3389/fpsyg.2021.611743

**Published:** 2021-06-18

**Authors:** Changmi Wang

**Affiliations:** The English Department, School of Foreign Language Studies, Zhejiang Sci-Tech University, Hangzhou, China

**Keywords:** English medium instruction, teaching self-efficacy, classroom language proficiency, language of instruction, language of interaction

## Abstract

This article examines the relationship between English medium instruction (EMI) teachers' classroom English proficiency and their teaching self-efficacy. The literature review highlights the difference between general language proficiency and classroom language proficiency by focusing on the EMI teachers' language of instruction and their language of interaction. Self-reported data were obtained using two measuring scales from 188 EMI teachers from a Chinese public university. The Pearson correlational analysis indicated that there was a strong positive relationship between the EMI teachers' classroom English proficiency and their teaching self-efficacy. Among the four constructs of the Classroom English Proficiency Scale, both language of instruction and language of interaction have a higher correlation with teaching self-efficacy than grammar or pronunciation. The linear regression analysis suggests that language of instruction has a significant contribution to the variance of teaching self-efficacy. The findings reveal the need to prioritize the strategic training of language of instruction skills to EMI teachers who are not so proficient in English. Arguably, this helps to foster the achievement and maintenance of higher teacher self-efficacy.

## Introduction

The use of English as a medium of instruction (EMI) has become increasingly common in non-native English-speaking countries (Costa and Coleman, 2013; Dearden, 2015, 2018; Macaro et al., 2018). English as a medium of instruction is the practice of using English to teach academic subjects in countries where the majority of the population's first language (L1) is not English. English as a medium of instruction teachers' limited English level has been reported in different cultural contexts (Doiz et al., 2013; Werther et al., 2014; Bradford, 2016). Arguably, EMI teachers' language challenge may result in their lower self-confidence in teaching (Wen et al., 2018; Pun and Thomas, 2020).

Much research has been conducted in the past two decades exploring the relationship between teachers' language proficiency and self-efficacy, a concept referring to teachers' beliefs in their teaching competence (Chacón, 2005; Eslami and Fatahi, 2008; Yilmaz, 2011). However, the previous studies failed to differentiate classroom language proficiency from general language proficiency. According to the language classification by Gierlinger (2013), there are four categories of “languages” in English-medium classroom teaching: general language, academic language, subject/domain specific language, and classroom language. English as a medium of instruction teachers' classroom language goes beyond general language proficiency (Elder and Kim, 2013; Macaro, 2020). In the same vein, Freeman (2017) contends that how EMI teachers' classroom language proficiency is described connects to the multi-dimensions of their teaching. The vague concept of “language proficiency” in the EMI context calls for a better understanding of the types of proficiency needed for EMI teachers' effective teaching (Macaro et al., 2021).

Literature on the EMI teachers' language assessment and certification practices provides valuable insights for understanding EMI teachers' classroom language proficiency. For example, in the Classroom Language Assessment (CLA) (Education Bureau of Hong Kong, 2011), the language of instruction and the language of interaction have been highlighted as two extra constructs. This differentiation in the description of teacher classroom language performance facilitates the conducting of further research on the relationship between EMI teachers' classroom English proficiency and their self-efficacy. In this study, EMI teachers' classroom English proficiency is also referred to as EMI teachers' classroom language proficiency, which is different from general language proficiency as will be discussed later.

This study sought to explore the relationship between EMI teachers' classroom English proficiency and their teaching self-efficacy. It differs from the previous studies in that EMI teachers' language measurement in this study is mainly conducted from the perspective of classroom language. This study is a quantitative correlational analysis, whose aim is to determine the extent to which classroom language proficiency contributes to teaching self-efficacy. Current language training practice for EMI teachers usually focuses on general language proficiency, which often cannot directly address EMI teachers' particular needs for classroom language improvement and self-efficacy development (Tsui, 2018). Therefore, the findings of this study help to generate an understanding of classroom language use in English medium instruction (EMI) and provide valuable insights for the development of EMI teachers' teaching self-efficacy.

## Literature Review

### Teacher Self-Efficacy

Self-efficacy, as a key concept in Bandura's Social Cognitive Theory (Bandura, 1997), refers to an individual's belief about their ability to accomplish a task. Although they are closely associated, self-efficacy is distinguished from self-confidence. Self-confidence, as a concept of personal traits in psychology, refers to an individual's self-judgment of their capabilities and skill to deal with the demands of various situations (Shrauger and Schohn, 1995). The term *self-efficacy* is preferred in educational research because self-confidence has no constructs or theoretical model used to define its determinants, processes, or effects (Klassen and Tze, 2014).

Tschannen-Moran et al. (1998) defined teacher efficacy as a teacher's view of their ability to successfully complete a defined teaching goal for a topic. Individuals who lack self-efficacy regarding their accomplishments are more likely to give up and limit their participation on tasks (Bandura, 1977; Brown, 1999). According to Tschannen-Moran and Hoy (2001), teaching self-efficacy may affect teachers' teaching performance, such as classroom behavior, teaching goals, efforts in teaching, and aspiration level. Furthermore, teachers' higher self-efficacy can work as a predictor of productive teaching and improve students' learning success (Goddard et al., 2004; Klassen and Tze, 2014).

### EMI Teachers' Classroom Language Proficiency

General language proficiency has been viewed as one of the key factors determining a teacher's successful implementation of English-medium instruction (Klaassen, 2008; Tange, 2010; Tatzl, 2011; Jensen et al., 2013; Werther et al., 2014; O'Dowd, 2018; Rose et al., 2019). Scholars tend to believe that a minimum level of English proficiency should be required for EMI teachers' successful teaching (Ball and Lindsay, 2013; Soren, 2013). The Common European Framework of Reference (CEFR) suggested that the level of C1 (proficient users) could meet the minimum proficiency level (tertiary) (Council of Europe, 2001; Klaassen and Bos, 2010). The C1 cut-off score corresponds to an internet-based test of TOEFL score of 83 or IELTS band of 6.5 (ETS, 2010; Lim et al., 2013). However, teachers' general English proficiency cannot guarantee their classroom language proficiency (Freeman et al., 2015).

The literature notes some valuable thinking concerning EMI teachers' classroom language from the perspective of English for Specific Purposes (ESP). English-speaking teachers need general language ability, academic language ability, and professional language knowledge to effectively teach (Elder, 1993; Cummins, 1994). It was concluded that having good general language proficiency is not enough for the teacher's classroom teaching (Freeman et al., 2015). The teacher's effective teaching not only needs proficiency in the language, but also some language skills such as giving instructions, questioning, and signaling (Cullen, 1994; Elder and Kim, 2013). According to Elder and Kim (2013), EMI teachers' CLA should adopt an approach that combines “a general proficiency screening tool” and “a more context-specific, performance-based measure” (p. 466). This approach emphasizes classroom language use for instruction, which goes beyond general language proficiency (Macaro et al., 2018).

In the past decade, EMI teachers' CLA practice appears to emphasize the teacher's English for lecturing and interacting. This tendency shows that the assessment focus is shifting from general language proficiency to the teacher's language use for teaching. Examples of the assessment practices include the Test of Oral English Proficiency for Academic Staff (TOEPAS) at the University of Copenhagen in Denmark (Kling and Stæhr, 2012), the English Medium Instruction Quality Management (EMI^QM^) at the University of Freiburg in Germany (Dubow and Gundermann, 2017), and the Language Proficiency Assessment for Teachers (English Language) (LPATE) in Hong Kong (Education Bureau of Hong Kong, 2011).

The TOEPAS (Kling and Stæhr, 2012) focuses on lecturing and teacher-student interactive communication activities, emphasizing the most significant communicative tasks such as presenting, explaining domain-specific terms and concepts, asking questions, and responding to student questions. Another assessment practice named EMI^QM^ aims to assure the quality of teacher language and to meet the needs of multicultural classrooms (Dubow and Gundermann, 2017). The evaluation criteria of the EMI^QM^ consist of two aspects: linguistic and communicative competencies (Dubow and Gundermann, 2017). Although these assessments have taken some measures to ensure reliability and validity, there seems to be no specific data reported in the publications.

Compared with the two assessments mentioned above, the LPATE (Education Bureau of Hong Kong, 2011) has been a more validated test. The LPATE, implemented in 2001, has become a benchmark for the Hong Kong government to ensure teachers' classroom language proficiency. According to the LPATE handbook (Education Bureau of Hong Kong, 2011), the assessment consists of five sections: reading, writing, listening, speaking, and the CLA. The CLA is distinct from other language ability assessments in that it includes two extra constructs: the language of instruction and the language of interaction.

### Language of Instruction and Language of Interaction

The concept of English as the medium of instruction implies that EMI teachers' language use is for teaching academic discipline knowledge. This functional nature of the language used in the EMI classroom may be reflected by the two constructs namely *language of instruction* and *language of interaction*, as classified by the CLA (Education Bureau of Hong Kong, 2011). As part of the Hong Kong LPATE assessment, the CLA assessment not only covers general language proficiency such as grammar and pronunciation, but also emphasizes the functional nature of classroom language use for instructing and interacting, which might be an appropriate approach to EMI teachers' classroom language measurement.

For a better understanding of EMI teachers' classroom language, it might be helpful to refer to the term *English-for-Teaching*, which has been recently discussed in teaching English as a foreign language (EFL) (Freeman et al., 2015). *English-for-Teaching* is defined as “the essential English language skills a teacher needs to be able to prepare and enact the lesson in a standardized (usually national) curriculum in English in a way that is recognizable and understandable to other speakers of the language” (Young et al., 2014, p. 5). The *English-for-Teaching* programme, with its aim to narrow the gap between general language proficiency and classroom language proficiency, provides skills training such as managing the classroom, lecturing, giving feedback, and assessing students' performance (Freeman et al., 2015). For successful classroom instruction and interaction, the EMI teacher should have not only language skills including domain specific terminology, but also the discourse competence for “effective classroom delivery of subject content” (Elder, 2001, p. 152).

### Relationship Between Language Proficiency and Teaching Self-Efficacy

The existing research on the relationship of language proficiency and self-efficacy has been mainly conducted among non-native EFL teachers (Chacón, 2005; Eslami and Fatahi, 2008; Yilmaz, 2011; Choi and Lee, 2016; Faez and Karas, 2017). Chacón's (2005) study found that self-efficacy in student engagement of the 104 EFL teachers in Venezuela was significantly correlated with all the four skills of speaking, listening, reading, and writing. Choi and Lee (2016) concluded that teachers' language proficiency and pedagogical capabilities are interdependent after investigating teachers' self-reported English proficiency and teaching efficacy in their English instruction. According to the meta-analysis by Faez and Karas (2017), studies of teaching EFL generally report a positive correlation between English teachers' self-reported language proficiency and their perceived self-efficacy.

Compared with the importance of general English proficiency for teaching in EFL, English for teaching is the functional nature of the teacher's classroom language use in the context of EMI. Previous studies conducted in the context of teaching EFL focusing on the relationship between teachers' English proficiency and self-efficacy cannot show whether and how EMI teachers' decreasing teaching self-efficacy is attributed to their language inadequacy (Werther et al., 2014; Chapple, 2015; Dearden, 2015). In addition, the previous studies failed to differentiate classroom language use from general language proficiency. Thus, these studies cannot provide valuable information for the development of EMI teachers' self-efficacy in sync with the notions of language of instruction and language of interaction.

This present study is guided by the following research questions:

(1) Is there any relationship between EMI teachers' classroom English proficiency and their teaching self-efficacy?
(2) Is there any relationship between EMI teachers' grammar, pronunciation, language of instruction, language of interaction, and their teaching self-efficacy?
(3) To what extent does EMI teachers' grammar, pronunciation, language of instruction, or language of interaction affect their teaching self-efficacy?

## Methods

### Data Collection

To answer the research questions, a quantitative questionnaire packet with two scales was used in this study. As discussed above, the scales for general English proficiency used in EFL may not be appropriate for EMI classroom language measurement. Thus, the Classroom English Proficiency Scale (see Appendix), adapted from the CLA of LPATE (Education Bureau of Hong Kong, 2011), was used to measure EMI teachers' classroom language proficiency. English as a medium of instruction teacher self-efficacy was measured via use of the Teachers' Sense of Efficacy Scale (TSES) (Tschannen-Moran and Hoy, 2001). The questionnaire used a 5-point Likert scale of 0 (strongly disagree) to 4 (strongly agree).

A convenience sampling method was used for recruiting participants. Participants for this study were EMI teachers at a Chinese public university. The EMI programmes of varying levels at this university all have a mixture of international students and local Chinese students. The participants were all native Chinese who use English as their classroom language. The link to the self-report survey was sent by email to the participating EMI teachers at the university.

The study was conducted in an ethically sound manner with participants being informed about their right to withdraw at any time without giving a reason. To guarantee the confidentiality of their responses, data were always kept confidential. Participants would not be identified in the publication. The G-power calculation (with an alpha of 0.05 and an effect size of 0.06) tells that a total sample size of 185 participants can reach a power of 0.8. Finally, a total of 188 teachers participated in the survey.

### Instruments

#### The Classroom English Proficiency Scale

The CLA examines four constructs: “grammatical and lexical accuracy and range; pronunciation, stress, and intonation; the language of interaction; and the language of instruction” (Education Bureau of Hong Kong, 2011, p. 80). Although there is no published indication on the reliability of the CLA component at this time, confidential access to the statistics reports that the CLA has “high validity” (Coniam and Falvey, 2013, p. 152). The reliability and validity for the adapted scale were checked using the SPSS (IBM, 2015).

The original application of this scale was through classroom observation. In this study, the scale was adapted into 12 self-reportable items, with a total of three statements per four components. The four components are grammatical and lexical accuracy and range; pronunciation, stress, and intonation; the language of interaction; and the language of instruction (Education Bureau of Hong Kong, 2011). The language of interaction includes eliciting, responding, and providing feedback; the language of instruction includes presenting, giving instructions, and signaling (Education Bureau of Hong Kong, 2011).

#### The Teachers' Sense of Efficacy Scale

The TSES by Tschannen-Moran and Hoy (2001) has been proved to be a reliable scale for measuring teaching self-efficacy. According to Tschannen-Moran and Hoy (2001), this scale includes three factors: self-efficacy for instructional strategies, for classroom management, and for student engagement. The reported reliabilities for the teacher efficacy sub-scales are 0.91 for instruction, 0.90 for management, and 0.87 for engagement, respectively (Tschannen-Moran and Hoy, 2001).

The TSES scale has two versions: the 24-item long form and the 12-item short form. This study used the short form, which was adjusted slightly for the university setting, with “children” changed into “students” and two items replaced by the ones from the long form. In the second factor of classroom management, “How much can you do to calm a student who is disruptive or noisy?” was replaced by “How well can you establish routines to keep activities running smoothly?” Similarly, “How much can you assist families in helping their children do well in school?” was replaced by “What can you do to help your students think critically?”

Additionally, the original questions were changed into statements to keep this scale in conformity with all the other scales. For example, the original question “To what extent can you craft good questions for your students?” was changed to “I can craft good questions for my student.” This allowed a Likert scale to be used across the whole survey.

### Reliability and Validity

The SPSS v. 23.0 (IBM, 2015) was used for reliability analysis and exploratory factor analysis. The internal consistency Cronbach α of the Classroom Language Scale is 0.94, and the Teacher Self-Efficacy Scale 0.90. The Total Variance Explained table and the Rotated Factor Matrix table for each scale indicated that roughly all the factors for the scales are consistent with the constructs of the original scales. This indicated that the scales have good construct validity.

## Results

### Is There Any Relationship Between EMI Teachers' Classroom English Proficiency, Its Components, and Their Teaching Self-Efficacy?

The descriptive statistics presented the means and standard deviations for all the scales and the sub-scales (Table 1). All the variables and the constructs in this study were near normal based on the Skewness and Kurtosis. By running the frequencies in SPSS (IBM, 2015), no data were missing. Correlational analysis and linear regression analysis were then conducted to examine the relationship between EMI teachers' classroom English proficiency and their teaching self-efficacy.

**Table 1:** Descriptive statistics for English proficiency, teaching self-efficacy, and their constructs (means, standard deviation, skewness, and kurtosis).

			**Skewness**		**Kurtosis**	
**Variables**	***M***	***SD***	***Statistic***	***SE***	***Statistic***	***SE***
English proficiency	2.84	0.63	−0.49	0.18	0.59	0.35
Teaching self-efficacy	2.97	0.46	0.06	0.18	0.18	0.35
Grammar	2.71	0.64	−0.15	0.18	-0.28	0.35
Pronunciation	2.80	0.76	−0.31	0.18	0.04	0.35
Language of instruction	2.99	0.71	−0.76	0.18	0.56	0.35
Language of interaction	2.87	0.75	−0.54	0.18	1.00	0.35
Self-efficacy for instructional strategies	3.01	0.60	−0.77	0.18	2.60	0.35
Self-efficacy for classroom management	3.07	0.52	0.01	0.18	0.53	0.35
Self-efficacy for student engagement	2.82	0.53	0.05	0.18	0.29	0.35

*N = 188. M, SD, and SE represent mean, standard deviation, and standard error, respectively. Five-point Likert scale where 0 = strongly disagree to 4 = strongly agree. The maximum score is 4*.

The correlational analysis was used to answer the first two research questions. The results of the Pearson correlation indicated that there was a strong positive correlation between the teacher's English proficiency and their teaching self-efficacy (*r* = 0.64, *p* < 0.001). Among the four components of the classroom English scale, *language of instruction* (*r* = 0.62, *p* < 0.001) and *language of interaction* (*r* = 0.59, *p* < 0.001) had a higher correlation with Teaching Self-efficacy, compared with *grammar* (*r* = 0.52, *p* < 0.001), and *pronunciation* (*r* = 0.52, *p* < 0.001) (Table 2).

**Table 2:** Pearson Correlations of teaching self-efficacy (SE) and the constructs of English proficiency (EP) (*N* = 188).

	**1**	**2**	**3**	**4**	**5**
Teaching self-efficacy (SE)	–				
Grammar	0.52	–			
Pronunciation	0.52	0.68	–		
Language of interaction	0.59	0.64	0.69	–	
Language of instruction	0.62	0.63	0.68	0.82	–

*p < 0.001*.

### To What Extent Does EMI Teachers' Grammar, Pronunciation, Language of Instruction, or Language of Interaction Affect Their Teaching Self-Efficacy?

To answer Question 3, a multiple regression analysis was first used whereby the four constructs of English Proficiency were regressed on Teaching Self-efficacy. The multiple regression determines the amount of variation in EMI teachers' teaching self-efficacy attributed to each of the four constructs of the classroom English proficiency as independent variables. In other words, the size of the coefficient for each construct gives the size of the effect that each construct has on EMI teachers' teaching self-efficacy.

The *R*^2^-value indicates how much the independent variables can explain the total variation in the dependent variable. In this case, the results of the regression indicated that EMI teachers' English proficiency explained 41% of the variance of their teaching self-efficacy [*R*^2^ = 0.41, *F*_(4, 183)_ = 33.48, *p* < 0.001] (Table 3). The results show that the overall level of *teaching self-efficacy* was significantly predicted by *language of instruction* (β = 0.33, *p* < 0.01), while *grammar, pronunciation, or language of interaction* had no significant contribution to the variance of *teaching self-efficacy* (Table 3). Thus, multicollinearity was taken into account, since the four constructs of English proficiency as independent variables are strongly correlated.

**Table 3:** Summary of multiple regression analysis for the constructs of English proficiency (EP) predicting teaching self-efficacy (SE) (*N* = 188).

						**Collinearity**	
**Variables**	***B***	***SE B***	**β**	***t***	***p***	**Tolerance**	**VIF**
(Constant)	1.61	0.12		12.99	0.00^*^		
Grammar	0.11	0.06	0.15	1.81	0.07	0.47	2.11
Pronunciation	0.05	0.05	0.08	0.92	0.36	0.41	2.41
Language of interaction	0.10	0.06	0.17	1.63	0.11	0.29	3.43
Language of instruction	0.22	0.07	0.33	3.22	0.00	0.30	3.35

*R^2^ = 0.41, F_(4, 183)_ = 33.48*,

* *p < 0.001*.

A stepwise linear regression was then conducted to resolve the multicollinearity between the four independent variables. The stepwise regression removed the redundant factors of *grammar, pronunciation*, and *language of interaction*. The optimized result showed that *language of instruction* explained 62% of the variance of EMI teachers' teaching self-efficacy [*R*^2^ = 0.62, *F*_(1, 186)_ = 114.50, *p* < 0.001].

## Discussion and Implications

### EMI Teachers' Classroom English Proficiency and Teaching Self-Efficacy

The results of this study show that there is a strong positive correlation between EMI teachers' classroom English proficiency and their teaching self-efficacy. This finding is consistent with the previous research in the field of teaching EFL, which found that teachers' general English proficiency plays an important role in teaching EFL (Chacón, 2005; Eslami and Fatahi, 2008; Yilmaz, 2011). This study highlights the function of classroom language, which helps with further in-depth analysis of the role of English as a medium of classroom instruction (Doiz and Lasagabaster, 2020).

The correlational analysis of teaching self-efficacy and the four constructs of classroom English show that the correlations of *language of instruction* and *language of interaction* with teaching self-efficacy are stronger than those of grammar and pronunciation. This suggests that general English proficiency has a weaker correlation with teaching self-efficacy, compared with language of interaction and language of instruction. The conceptualization of *language for instruction* seems to be meaningful for EMI teacher's language training (Margić and Vodopija-Krstanović, 2018). General language training for teachers' language proficiency improvement may not be sufficient in meeting teachers' professional development needs of effective teaching, that is, facilitating students' learning of different academic disciplines in the classroom (Freeman, 2017; Macaro et al., 2020).

In this study, the findings of stronger correlations of EMI teachers' classroom English proficiency with teaching self-efficacy provided additional evidence supporting the rationale behind the *English-for-Teaching* training programme (Freeman, 2017). It is worth noting that both the language of instruction and the language of interaction define the EMI teachers' classroom language proficiency. The results resonate with findings from recent research implemented in Japan and Vietnam (Freeman, 2017; Nhung, 2017). According to Nhung's (2017) study, classroom language training was perceived to be more practical for Vietnamese teachers' needs, compared with general language training. Therefore, this study supports the need to establish an EMI language training and certification programme with the focus on language of instruction and language of interaction.

### Language of Instruction and Teacher Self-Efficacy

The results of this study show that language of instruction is a significant predictor that influences EMI teachers' teaching self-efficacy, compared with grammar, pronunciation, and language of interaction. That is, the improvement of EMI teachers' language of instruction is more likely to lead to their higher teaching self-efficacy. This finding may have vital implications for the development and training of EMI teachers. To effectively increase EMI teachers' teaching self-efficacy, the English language development could focus on English for teaching, that is, the ability to implement effective classroom instruction. For example, the teacher might be taught how to use fixed sentence structures to explain concepts, terms, or lesson content, and how to give clear instructions in English when conducting activities, giving homework, and managing the classroom. In addition, appropriate English signals to indicate stages of a lesson might also be strengthened in the language training. Overall, this study provided evidence to suggest that the strategic training of language of instruction should be prioritized as a language training option in situations where EMI teachers are not so proficient in English to achieve and maintain higher teacher self-efficacy.

### Language of Interaction and Teacher Self-Efficacy

This study's results revealed that language of interaction, like grammar and pronunciation, had no significant contribution to the variance of teaching self-efficacy. This may be partly due to its collinearity with language of instruction, which was shown in the stepwise regression. Another possible explanation is that the participating teachers may tend to use the teacher-centered approach emphasizing more lecturing and being less interactive in the EMI classroom. On one hand, the participating EMI teachers in this study also had the challenges of limited classroom language proficiency. On the other hand, the students might tend to be less interested in participating in classroom interaction due to their inadequate command of English (Airey and Linder, 2006). However, there is no solid evidence to indicate that more lecturing would give the teacher a misconception that language of instruction is more important for their teaching success.

The result that language of interaction is not a significant predictor of teaching self-efficacy should be cautiously considered. It does not mean that language of interaction is not important for EMI teachers' teaching self-efficacy; on the contrary, it matters, considering the strong correlation mentioned above. Self-efficacy for student engagement probably entails adequate level of language of interaction; for successful classroom interaction, the teacher needs to use appropriate English to ask questions or to provide clues and hints, to respond to students' questions, such as seeking clarification, giving confirmation, and asking for repetition, and to give feedback skilfully in English, such as acknowledging, evaluating, and commenting on students' responses.

The inadequacy of classroom interaction can be evidenced by the results of the descriptive analysis of this study. The results show that the mean of language of interaction (*M* = 2.87, *SD* = 0.75) is much lower than that of the language of instruction (*M* = 2.99, *SD* = 0.71) (Table 1); the mean of Self-efficacy for student engagement (*M* = 2.82, *SD* = 0.53) is also the lowest among the components of teaching self-efficacy, compared with Self-efficacy for instructional strategies (*M* = 3.01, *SD* = 0.60) and Self-efficacy for classroom management (*M* = 3.07, *SD* = 0.52) (Table 1). These results suggest that the situation that some of the participating EMI teachers have experienced challenges in classroom interaction does exist. The lower scores of EMI teachers' language of interaction may provide an acceptable explanation for the findings of inadequate interaction noted in the literature (Doiz et al., 2013; Hu et al., 2014; Werther et al., 2014; Chapple, 2015; Wen et al., 2018; Lu and Dearden, 2021). This study also suggests that improving EMI teachers' language of interaction may be a possible approach to enhance their interactional competence (Llinares and Mendikoetxea, 2020). Arguably, how the EMI teacher perceives the importance of language of instruction and language of interaction still needs further research.

## Limitations and Future Research

This study has some limitations. The first limitation is the sample size. The participants were the EMI teachers from only one university, so it is unclear whether data from other samples in a different university, especially from a different country with a different pedagogical approach, would yield the same results. For example, the classroom culture in Chinese universities was once dominantly teacher-centered, while students at a European university may favor a learner-centered classroom culture. Future research needs to investigate an expanded population.

The second limitation is that data was self-reported rather than acquired by means of actual classroom observation. Self-reported data may not be able to fully represent teachers' classroom language performance. Future research may collect data by classroom observation, which would shed more light on the language in EMI classes.

Lastly, longitudinal studies following teachers through their training could be instructive. With the insights of this study, future language training may enhance the teacher's language of instruction and language of interaction. Thus, exciting possibilities lie ahead as longitudinal studies could bring to light more information about the significant effects of classroom language development.

## Data Availability Statement

The raw data supporting the conclusions of this article will be made available by the authors, without undue reservation.

## Ethics Statement

The studies involving human participants were reviewed and approved by The ethics committee of Zhejiang Sci-Tech University. Written informed consent for participation was not required for this study in accordance with the national legislation and the institutional requirements.

## Author's Note

Special permission was obtained from the copyright owners for the Teachers' Sense of Efficacy Scale (TSES) (Tschannen-Moran and Hoy, 2001).

## Author Contributions

The author confirms being the sole contributor of this work and has approved it for publication.

## Conflict of Interest

The author declares that the research was conducted in the absence of any commercial or financial relationships that could be construed as a potential conflict of interest.

## Appendix

**The Classroom English Proficiency Scale**

Use the following ratings:

*0* = *strongly disagree 1* = *disagree 2* = *uncertain 3* = *agree 4* = *strongly agree*

1. I can lecture with correct English grammatical structures.
2. I can use a broad range of English vocabulary.
3. I can use accurate words to express ideas.
4. I can speak English clearly with no systematic errors in pronunciation.
5. I know how to stress content words in pronunciation.
6. I can use intonation naturally to convey meaning.
7. I can use appropriate English to ask questions or to provide clues and hints.
8. I can use appropriate English to respond to students' questions, such as seeking clarification, giving confirmation, and asking for repetition.
9. I can give feedback skillfully in English, such as acknowledging, evaluating, and commenting on students' responses.
10. I can explain concepts, terms, or lesson content in clear English.
11. I can give clear instructions in English when conducting activities, giving homework, and managing the classroom.
12. I can use appropriate English signals (e.g., first, second, next) to indicate stages of a lesson.

**Note**: Grammatical and lexical accuracy and range items are 1, 2, and 3; Pronunciation, stress, and intonation items are 4, 5, and 6; The language of interaction items are 7, 8, and 9; The language of instruction items are 10, 11, and 12.

## References

[B1] Airey J. Linder C. (2006). Language and the experience of learning university physics in Sweden. Eur. J. Phys. 27, 553–560. 10.1088/0143-0807/27/3/009

[B2] Ball P. Lindsay D. (2013). Language demands and support for English-medium instruction in tertiary education: learning from a specific context, in English-Medium Instruction at Universities: Global Challenges, eds Doiz A. Lasagabaster D. Sierra J. M. (Bristol: Multilingual Matters), 44–64.

[B3] Bandura A. (1977). Self-efficacy: toward a unifying theory of behavioral changes. Psychol. Rev. 84, 191–215. 10.1037/0033-295X.84.2.191 847061

[B4] Bandura A. (1997). Self-Efficacy: The Exercise of Control. New York: Freeman.

[B5] Bradford A. (2016). Toward a typology of implementation challenges facing English-medium instruction in higher education: evidence from Japan. J. Stud. Int. Educ. 20, 339–356. 10.1177/1028315316647165

[B6] Brown B. L. (1999). Self-efficacy beliefs and career development. ERIC Digest 205, 1–8. Columbus, OH: ERIC Clearinghouse on Adult Career and Vocational Education. Available online at: ERIC database (ED429187).

[B7] Chacón C. (2005). Teachers' perceived efficacy among English as a foreign language teachers in middle schools in Venezuela. Teach. Teach. Educ. 21, 257–272. 10.1016/j.tate.2005.01.001

[B8] Chapple J. (2015). Teaching in English is not necessarily the teaching of English. Int. Educ. Stud. 8, 1–13. 10.5539/ies.v8n3p1

[B9] Choi E. Lee J. (2016). Investigating the relationship of target language proficiency and self-efficacy among nonnative EFL teachers. System 58, 49–63. 10.1016/j.system.2016.02.010

[B10] Coniam D. Falvey P. (2013). Ten years on: the Hong Kong language proficiency for teachers of English (LPATE). Lang. Test. 30, 147–155. 10.1177/0265532212459485

[B11] Costa F. Coleman J. A. (2013). A survey of English-medium instruction in Italian higher education. Int. J. Biling. Educ. Bilingual. 16, 3–19. 10.1080/13670050.2012.676621

[B12] Council of Europe. (2001). Common European *Framework* of *Reference* for *Languages*: Learning, *Teaching, Assessment*. Cambridge: Cambridge University Press.

[B13] Cullen R. (1994). Incorporating a language improvement component in teacher training programmes. ELT J. 48, 162–172. 10.1093/elt/48.2.162

[B14] Cummins J. (1994). Knowledge, power, and identity in teaching English as a second language, in Educating Second Language Children: The Whole Child, the Whole Curriculum, the Whole Community, ed Genesee F. (New York: Cambridge University Press), 33–58.

[B15] Dearden J. (2015). English as a Medium of Instruction – A Growing Global Phenomenon. British Council. Available online at: https://www.britishcouncil.org/sites/default/files/e484~_emi_cover_option_3_final_web.pdf (accessed December 10, 2018).

[B16] Dearden J. (2018). The changing roles of EMI academics and English language specialists, in Key Issues in English for Specific Purposes in Higher Education. English Language Education, Vol. 11, eds Kirkgöz Y. Dikilitaş K. (Cham: Springer), 323–338.

[B17] Doiz A. Lasagabaster D. (2020). Dealing with language issues in English-medium instruction at university: a comprehensive approach. Int. J. Biling. Educ. Bilingual. 23, 257–262. 10.1080/13670050.2020.1727409

[B18] Doiz A. Lasagabaster D. Sierra J. M. (eds.). (2013). English-Medium Instruction at Universities: Global Challenges. Bristol: Multilingual Matters.

[B19] Dubow G. Gundermann S. (2017). Certifying the linguistic and communicative competencies of teachers in English-medium instruction programmes. Lang. Learn. High. Educ. 7, 475–487. 10.1515/cercles-2017-0021

[B20] Education Bureau of Hong Kong. (2011). Language Proficiency Assessment for Teachers (English Language) Handbook. Available online at: https://www.edb.gov.hk (accessed December 10, 2018).

[B21] Elder C. (1993). Language proficiency as predictor of performance in teacher education. Melb. Pap. Lang. Test. 2, 1–17.

[B22] Elder C. (2001). Assessing the language proficiency of teachers: are there any border control? Lang. Test. 18, 149–170. 10.1177/026553220101800203

[B23] Elder C. Kim S. H. O. (2013). Assessing teachers' language proficiency, in The Companion to Language Assessment, Vol. 1, ed Kunnan A. (Malden, MA: Wiley-Blackwell), 454–470.

[B24] Eslami Z. R. Fatahi A. (2008). Teachers' sense of self-efficacy, English proficiency and instructional strategies: a study of nonnative EFL teachers in Iran. TESL-EJ 11, 1–19.

[B25] ETS. (2010). ETS Linking TOEFL iBT™ *Scores to IELTS® Scores – A Research Report*. Available online at: https://www.ets.org/s/toefl/pdf/linking_toefl_ibt_scores_to_ielts_scores.pdf (accessed December 10, 2018).

[B26] Faez F. Karas M. (2017). Connecting language proficiency to (self-reported) teaching ability: a review and analysis of research. RELC J. 48, 135–151. 10.1177/0033688217694755

[B27] Freeman D. (2017). The case for teachers' classroom English proficiency. RELC J. 48, 31–52. 10.1177/0033688217691073

[B28] Freeman D. Katz A. Gomez P. G. Burns A. (2015). English-for-teaching: rethinking teacher proficiency in the classroom. ELT J. 69, 129–139. 10.1093/elt/ccu074

[B29] Gierlinger E. (2013). The Languages for CLIL Model. Available online at: http://clilingmesoftly.wordpress.com/clil-teachers-tl-competence (accessed December 10, 2018).

[B30] Goddard R. D. Hoy W. K. Hoy A. W. (2004). Collective efficacy beliefs: theoretical developments, empirical evidence, and future directions. Educ. Res. 33, 3–13. 10.3102/0013189X033003003

[B31] Hu G. Li L. Lei J. (2014). English-medium instruction at a Chinese University: rhetoric and reality. Lang. Policy, 13, 21–40. 10.1007/s10993-013-9298-3

[B32] IBM. (2015). Statistical Package for Social Sciences (Windows, Version 23) [Computer Software]. IBM.

[B33] Jensen C. Denver L. Mees I. M. Werther C. (2013). Students' attitudes to lecturers' English in English-medium higher education in Denmark. Nord. J. Engl. Stud. 12, 87–112. 10.35360/njes.277

[B34] Klaassen R. G. (2008). Preparing lecturers for English-medium instruction, in Realizing Content and Language Integration in Higher Education, eds Wilkinson R. Zegers V. (Maastricht: Maastricht University), 32–42.

[B35] Klaassen R. G. Bos M. (2010). English language screening for scientific staff at Delft University of Technology. HERMES 23, 61–75. 10.7146/hjlcb.v23i45.97347

[B36] Klassen R. M. Tze V. M. (2014). Teachers' self-efficacy, personality, and teaching effectiveness: a meta-analysis. Educ. Res. Rev. 12, 59–76. 10.1016/j.edurev.2014.06.001

[B37] Kling J. Stæhr L. S. (2012). The Development of the Test of Oral English Proficiency for Academic Staff (TOEPAS). Centre for Internationalization and Parallel Language Use (CIP), University of Copenhagen. Available online at: http://cip.ku.dk/forskning/cip_publikationer/CIP_TOPEPAS_Technical_Report.pdf (accessed December 10, 2018).

[B38] Lim G. S. Geranpayeh A. Khalifa H. Buckendahl C. W. (2013). Standard setting to an international reference framework: implications for theory and practice. Int. J. Test. 13, 32–49. 10.1080/15305058.2012.678526

[B39] Llinares A. Mendikoetxea A. (2020). Enhancing interactional competence in EMI: teacher reflective practices, in Teacher Training for English-Medium Instruction in Higher Education, ed del Mar Sánchez-Pérez M. (Madraid: IGI Global), 87–105.

[B40] Lu Z. Dearden J. (2021). Professional development in English medium instruction in higher education: a case study, in Teaching and Researching Chinese EFL/ESL Learners in Higher Education, 1st Edn., eds Lu Z. Liu M. Zhang W. (London: Routledge), 59–80.

[B41] Macaro E. (2020). Exploring the role of language in English medium instruction. Int. J. Biling. Educ. Bilingual. 23, 263–276. 10.1080/13670050.2019.1620678

[B42] Macaro E. Akincioglu M. Han S. (2020). English medium instruction in higher education: teacher perspectives on professional development and certification. Int. J. Appl. Linguist. 30, 144–157. 10.1111/ijal.12272

[B43] Macaro E. Curle S. Pun J. An J. Dearden J. (2018). A systematic review of English medium instruction in higher education. Lang. Teach. 51, 36–76. 10.1017/S0261444817000350

[B44] Macaro E. Sahan K. Rose H. (2021). The profiles of English medium instruction teachers in higher education. Int. J. Appl. Linguist. 2021, 1–17. 10.1111/ijal.12344

[B45] Margić B. D. Vodopija-Krstanović I. (2018). Language development for English-medium instruction: teachers' perceptions, reflections and learning. J. Engl. Acad. Purp. 35, 31–41. 10.1016/j.jeap.2018.06.005

[B46] Nhung P. T. H. (2017). General English proficiency or English for teaching? The preferences of in-service teachers. RELC J. 49:0033688217691446. 10.1177/0033688217691446

[B47] O'Dowd R. (2018). The training and accreditation of teachers for English medium instruction: an overview of practice in European universities. Int. J. Biling. Educ. Bilingual. 21, 553–563. 10.1080/13670050.2018.1491945

[B48] Pun J. K. Thomas N. (2020). English medium instruction: Teachers' challenges and coping strategies. ELT J. 74, 247–257. 10.1093/elt/ccaa024

[B49] Rose H. Curle S. Aizawa I. Thompson G. (2019). What drives success in English medium taught courses? The interplay between language proficiency, academic skills, and motivation. Stud. High. Educ. 45, 2149–2161. 10.1080/03075079.2019.1590690

[B50] Shrauger J. S. Schohn M. (1995). Self-confidence in college students: conceptualization, measurement, and behavioral implications. Assessment 2, 255–278. 10.1177/1073191195002003006

[B51] Soren J. K. (2013). Teacher Identity in English-Medium Instruction: Teacher Cognitions from a Danish Tertiary Education Context. Doctoral dissertation, University of Copenhagen. Available online at: https://tel.archives-ouvertes.fr/tel-00863816/document (accessed December 10, 2018).

[B52] Tange H. (2010). Caught in the Tower of Babel: university lecturers' experiences with internationalisation. Lang. Intercul. Commun. 10, 137–149. 10.1080/14708470903342138

[B53] Tatzl D. (2011). English-medium masters' programmes at an Austrian university of applied sciences: attitudes, experiences and challenges. J. Engl. Acad. Purp. 10, 252–270. 10.1016/j.jeap.2011.08.003

[B54] Tschannen-Moran M. Hoy A. W. (2001). Teacher efficacy: capturing an elusive construct. Teach. Teach. Educ. 17, 783–805. 10.1016/S0742-051X(01)00036-1

[B55] Tschannen-Moran M. Hoy A. W. Hoy W. K. (1998). Teacher efficacy: its meaning and measure. Rev. Educ. Res. 68, 202–248. 10.3102/00346543068002202

[B56] Tsui C. (2018). Teacher efficacy: a case study of faculty beliefs in an English-Medium Instruction teacher training program. Taiwan J. TESOL 15, 101–128. 10.30397/TJTESOL.201804_15(1).0004

[B57] Wen W. Hu D. Hao J. (2018). International students' experiences in China: does the planned reverse mobility work? Int. J. Educ. Dev. 61, 204–212. 10.1016/j.ijedudev.2017.03.004

[B58] Werther C. Denver L. Jensen C. Mees I. M. (2014). Using English as a medium of instruction at university level in Denmark: the lecturer's perspective. J. Multiling. Multicult. Dev. 35, 443–462. 10.1080/01434632.2013.868901

[B59] Yilmaz C. (2011). Teachers' perceptions of self-efficacy, English proficiency, and instructional stategies. Soc. Behav. Pers. 39, 91–100. 10.2224/sbp.2011.39.1.91

[B60] Young J. W. Freeman D. Hauck M. C. Gomez P. G. Papageorgiou S. (2014). A Design Framework for the ELTeach Program Assessments. ETS Research Report No. RR-14–36, Educational Testing Service. 10.1002/ets2.12036

